# High Bodyweight Variability Increases Depression Risk in Patients With Type 2 Diabetes Mellitus: A Nationwide Cohort Study in Korea

**DOI:** 10.3389/fpsyt.2021.765129

**Published:** 2021-12-10

**Authors:** Ji Hyun An, Kyung-do Han, Jin-Hyung Jung, Juhwan Yoo, Maurizio Fava, David Mischoulon, Su-Min Jung, Dong Wook Shin, Kyu Yeon Hur, Hong Jin Jeon

**Affiliations:** ^1^Department of Psychiatry, Depression Center, Samsung Medical Center, Sungkyunkwan University School of Medicine, Seoul, South Korea; ^2^Department of Statistics and Actuarial Science, Soongsil University, Seoul, South Korea; ^3^Department of Biomedicine and Health Science, The Catholic University of Korea, Seoul, South Korea; ^4^Department of Biostatics, College of Medicine, The Catholic University of Korea, Seoul, South Korea; ^5^Depression Clinical and Research Program, Massachusetts General Hospital, Harvard Medical School, Boston, MA, United States; ^6^Department of Family Medicine/Supportive Care Center, Samsung Medical Center, Seoul, South Korea; ^7^Division of Endocrinology and Metabolism, Department of Medicine, Samsung Medical Center, Sungkyunkwan University School of Medicine, Seoul, South Korea; ^8^Department of Health Sciences and Technology, Samsung Advanced Institute for Health Sciences and Technology (SAIHST), Sungkyunkwan University, Seoul, South Korea; ^9^Department of Medical Device Management and Research, Samsung Advanced Institute for Health Sciences and Technology (SAIHST), Sungkyunkwan University, Seoul, South Korea; ^10^Department of Clinical Research Design and Evaluation, Samsung Advanced Institute for Health Sciences and Technology (SAIHST), Sungkyunkwan University, Seoul, South Korea

**Keywords:** diabetes mellitus, depression, body weight variability, nationwide, body weight

## Abstract

**Objectives:** Although obesity is associated with increased risk for depression in patients with type 2 diabetes mellitus (DM), the relationship between body weight variability (BWV) and depression remains poorly studied. This study was to investigate the incidence of depression in patients with type 2 DM according to their BWV.

**Methods:** Intraindividual variation in body weight were measured in the nationwide, population-based retrospective cohort of 540,293 patients with type 2 DM from the Korean national health insurance system between 2009 and 2010. The diagnoses of new-onset depression occurring until the end of 2017 were ascertained. Risk of new-onset depression was examined using multivariate-adjusted Cox proportional hazards regression analysis by BWV quartile.

**Results:** 93,149 (17.2%) patients developed new-onset depression for the follow up. BWV was significantly associated with an increased risk of depression after adjusting for confounding factors. The highest BWV quartile group had a hazard ratio (HR) of 1.17 (95% CI 1.15–1.19) compared to the lowest BWV quartile group as a reference. Obese patients in the highest BWV quartile group showed 12% increased risk of depression (HR 1.12, 95% CI 1.09–1.15) while non-obese patients in the highest BWV quartile group showed 20% increased risk of depression (HR: 1.20, 95% CI: 1.17–1.23) compared to their respective lowest BWV quartile groups.

**Conclusion:** A higher BWV was significantly associated with an increased risk of depression in patients with type 2 DM. Thus, BWV may serve as an indicator for early detection of depression in type 2 DM patients.

## Introduction

For patients with type 2 diabetes mellitus (DM), weight control through lifestyle intervention is important since obesity is associated with poor metabolic outcomes such as increased blood pressure, poor glycemic control, and worsening lipid parameters (1, 2). Furthermore, there is also growing evidence that body weight variability (BWV) is associated with an increased risk of cardiovascular events or mortality in patients with DM (3–5). About 20–30% of patients with type 2 DM suffer from depression (6, 7). The presence of comorbid depression in type 2 DM patients can increase the disease burden and worsen the prognosis (8–11). This has been hypothesized to be related to an increase in allostatic load (12). In addition, obesity has been suggested as a major etiological factor to developing depression in persons with type 2 DM (13, 14), through mechanisms such as autonomic/neuroendocrine dysregulation, pro-inflammatory state, or brain structural alterations (8, 15). This suggests that the metabolic pathway might be involved in the occurrence of depression in diabetic patients. Beside obesity, BWV also occurs more often in DM patients than in the general population due to lifestyle preference (exercise, dietary habit. etc.), therapeutic intervention (antidiabetic medication), or the course of the disease (weight loss due to insufficient insulin, weight gain as a premorbid status of DM, etc.). Thus, a relationship between BWV and poor metabolic outcomes can be easily inferred, this relationship affecting psychological outcomes and body weight of diabetic patients.

Several studies have reported the association between BWV and poor mental health outcome (16–18). However, whether BWV is associated with the development of incident depression in type 2 DM is unknown. Therefore, the objective of this nationwide population study was to examine the association between BWV and development of incident depression in type 2 DM patients. Investigating the impact of intra-individual BWV would give a better understanding of depression etiology than simply measuring body weight itself in diabetic patients. We hypothesized that a higher BWV would be associated with an increased risk of depression in type 2 DM.

## Methods

### Data Collection

This study was conducted using the dataset of the Korean national health insurance system (NHIS), the single insurer managed by the Korean government. The NHIS contains complete health information of about 50 million people (97% of Korean population), including sociodemographic data, clinical diagnosis with International Classification of Disease-10 Clinical modification (ICD-10-CM) codes, treatments, and health service utilization as described elsewhere (19). The NHIS also provides national health examination programs to Koreans aged over 40 years biennially or annually to workers regardless of age to examine individual health behaviors and obtain laboratory results and anthropometric measurements. We used NHIS health data and health examinations in the study.

### Study Population

**Figure 1:**
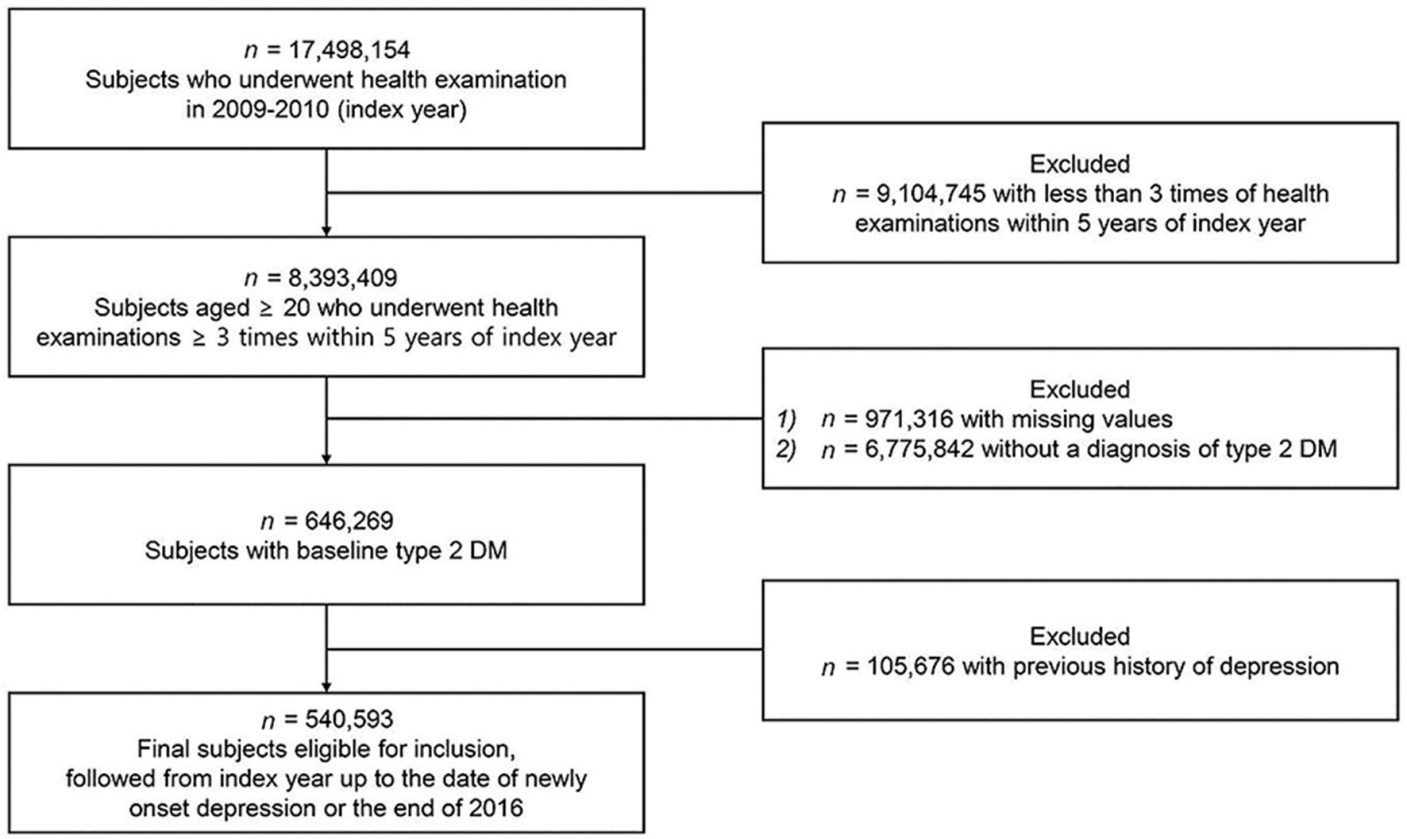
Flow chart showing the selection of study subjects. The target population 17,498,154 represents about one-fifth of the country's population. Type 2 DM patients were defined as those who had ICD-10-CM codes of E11-E14 with at least one prescription of antidiabetic medication or with a fasting glucose level ≥ 126 mg/dL.

Among 17,498,154 patients who underwent medical examinations during the index period (from January 1, 2009 to December 31, 2010), 8,393,409 patients who completed three or more medical examinations within 5 years prior to the index period were chosen. Type 2 DM patients were defined as those who had ICD-10-CM codes of E11–E14 with at least one prescription of antidiabetic medication or with a fasting glucose level ≥ 126 mg/dL in laboratory results. Finally, 540,590 patients aged 30 years or more with type 2 DM were identified after excluding those with missing data (*n* = 971,316) and those with a previous history of depression or new depression diagnosis within 2 years of the index year (*n* = 105,676) (Figure 1). All study patients were followed up until December 31, 2016. The mean duration of follow up was 5.42 ± 1.53 years. The entire study process was approved by the Institutional Review Board (IRB) of Samsung Medical Center in Seoul, Korea (IRB No: 2020-10-034). Informed consent was waived since this research did not involve any identifiable private information of study patients.

### Study Outcome: Diagnosis of Depression

The primary outcome of this study was new-onset depression defined as ICD-10-CM codes for depression (F32.0-32.9 for major depressive disorder with single episode and F 33.0-33.9 for major depressive disorder with recurrent episodes). Study patients were followed up for new-onset depression with a 2 year time lag after the index date until the diagnosis of new-onset depression, censoring by death or the end of the study (December 31, 2017).

### Definition of Obesity and Indices of Body Weight Variability

Obesity was defined as BMI ≥ 25 kg/m^2^ based on the World Health Organization (WHO) recommendations for Asians (20). Three or more body weight measurements obtained within 5 years of the index period were used to calculate individual BWV. Four indices, including standard deviation (SD), coefficient of variation (CV), average successive variability (ASV) (21), and variability independent of the mean (VIM), were used to measure BWV. VIM was used as the primary measure of BWV, which was calculated as 100 × SD/mean^β^, where β was the regression coefficient based on the natural logarithm of SD on the natural logarithm of the mean (22).

### Definition of Covariates

Baseline sociodemographic and lifestyle data included age, sex, income level (under 20% or above), current smoking status, alcohol consumption (≥ 30 g of alcohol per day indicating heavy drinking) (23), and regular exercise (vigorous physical activity three times or more per week or moderate physical activity five times or more per week) (24). A list of standardized health examination data such as height, weight, waist circumference, fasting glucose, blood pressure (systolic and diastolic), total cholesterol, high-density lipoprotein (HDL) cholesterol, low-density lipoprotein (LDL) cholesterol, triglycerides, and creatinine level was also included as covariates.

Baseline medical comorbidities were defined as a combination of laboratory measurement or ICD-10-CM diagnosis and prescribed medications (25, 26). Hypertension was defined as SBP/DBP ≥ 140/90 mmHg or ICD-10-CM code I10-13 or I15 with at least one prescription claim of antihypertensive medication per year. Dyslipidemia was defined as total cholesterol level ≥ 240 mg/dL or an ICD-10-CM code of E78 with at least one prescription claim of lipid lowering medication per year. Chronic kidney disease was defined as estimated glomerular filtration rate (eGFR) <60 mL/min/1.73 m (27). Myocardial infarction (MI) was defined as ICD-10-CM code I12-22. Ischemic stroke was defined as ICD-10-CM I63-64 and evidence in brain CT or MRI. Atrial fibrillation was defined as ICD-10-CM I48. Malignancy was defined as ICD-10-CM C00-96 and exempted calculation code for severe disease (28). All covariates were assessed at the index date.

### Statistical Analysis

Baseline sociodemographic and lifestyle data, health examination data, and medical comorbidities were presented in relation to newly developed depression. We performed analysis of variance (ANOVA) for continuous variables and χ^2^ test for categorical variables. Patients were allocated into four groups according to BWV quartile of VIM. The risk of depression for each of the three higher quartiles of BWV (Q2, Q3, and Q4) relative to the lowest quartile (Q1) was calculated by Cox proportional hazards regression analysis after adjusting for age, sex, smoking status, drinking status, level of physical activity, low income, presence of medical comorbidities including hypertension, dyslipidemia, and variables related to severity of diabetes including insulin use, number of oral anti-diabetic medications, and duration of diabetes. Further analyses examined the relationship between BWV and risk of depression according to presence of baseline obesity to explore whether obese patients experiencing BWV were at higher risk for developing depression than non-obese patients. Kaplan-Meier estimates for incidence probability of depression were presented for BWV of each quartile group. Stratified analyses were performed for potential modification effects by age strata, sex, medical comorbidities, and variables related to the severity of diabetes. Hazard ratio (HR) of depression in stratified analyses was calculated for the highest quartile group (Q4) of BWV compared to lower quartile groups (Q1–Q3). All statistical analyses were conducted with Statistical Analysis System (SAS) version 9.4 (SAS Institute Inc., Cary, NC, USA). An alpha level of 0.05 indicated statistical significance.

## Results

### Baseline Characteristics

Of 540,593 type 2 DM patients included in this cohort, 93,149 patients developed depression during the follow-up period (mean = 5.41 ± 1.53 years). Baseline sociodemographic and lifestyle differences between patients with and without new-onset depression are shown in Table 1. Type 2 DM patients with depression were older, more likely to be female, less likely to be smokers, and less likely to drink. They had a lower income but higher prevalence of medical comorbidities including hypertension, dyslipidemia, cardiovascular diseases, cancer, and chronic kidney disease. Type 2 DM patients with depression also had lower rate of obesity but higher BWV than those without depression.

**Table 1:** Baseline characteristics of study patients according to the presence of new-onset depression.

**Mean ± SD or *N*, (%)**	**Total**	**Depression**		***p*-Value**
	**(*N* = 540593)**			
		**No**	**Yes**	
		**(*N* = 447444)**	**(*N* = 93149)**	
Sex (male)	371,779 (68.77)	320,742 (71.68)	51,037 (54.79)	<0.0001
Age (years)	57.03 ± 11.8	55.94 ± 11.72	62.25 ± 10.74	<0.0001
Current smoker	141,735 (26.22)	124,353 (27.79)	17,382 (18.66)	<0.0001
Heavy alcohol drinker	54,522 (10.09)	47,345 (10.58)	7,177 (7.7)	<0.0001
Regular exerciser	130,122 (24.07)	108,893 (24.34)	21,229 (22.79)	<0.0001
Low income	99,391 (18.39)	80,698 (18.04)	18,693 (20.07)	<0.0001
Height (cm)	163.63 ± 8.95	164.24 ± 8.81	160.69 ± 9.02	<0.0001
Weight (kg)	67.09 ± 11.36	67.68 ± 11.39	64.22 ± 10.77	<0.0001
BMI (kg/m^2^)	24.97 ± 3.15	25.01 ± 3.15	24.8 ± 3.16	<0.0001
Obesity (BMI ≥ 25)	254,124 (47.01)	212,181 (47.42)	41,943 (45.03)	<0.0001
VIM of weight	1.96 ± 1.43	1.93 ± 1.41	2.06 ± 1.53	<0.0001
ASV of weight	2.3 ± 1.8	2.28 ± 1.79	2.37 ± 1.85	<0.0001
SD of weight	2.02 ± 1.49	2.02 ± 1.48	2.05 ± 1.52	<0.0001
CV of weight	3.04 ± 2.23	3 ± 2.19	3.22 ± 2.41	<0.0001
**Medical comorbidities**				
Hypertension	301,051 (55.69)	242,417 (54.18)	58,634 (62.95)	<0.0001
Dyslipidemia	215,389 (39.84)	174,236 (38.94)	41,153 (44.18)	<0.0001
Myocardial infarction	5,867 (1.09)	4,359 (0.97)	1,508 (1.62)	<0.0001
Ischemic stroke	20,728 (3.83)	13,821 (3.09)	6,907 (7.42)	<0.0001
Atrial fibrillation	6,199 (1.15)	4,492 (1)	1,707 (1.83)	<0.0001
Cancer	20,170 (3.73)	15,574 (3.48)	4,596 (4.93)	<0.0001
Chronic kidney disease	59,244 (10.96)	45,750 (10.22)	13,494 (14.49)	<0.0001
Systolic BP (mmHg)	128.68 ± 15.1	128.62 ± 15.02	128.99 ± 15.48	<0.0001
Diastolic BP (mmHg)	79.07 ± 9.84	79.22 ± 9.84	78.32 ± 9.84	<0.0001
eGFR (mL/min/1.73m^2^)	83.74 ± 36.99	84.17 ± 37.39	81.71 ± 34.91	<0.0001
HDL cholesterol (mg/dL)	51.54 ± 21.22	51.44 ± 21.01	51.98 ± 22.2	<0.0001
LDL cholesterol (mg/dL)	110.78 ± 44.99	110.86 ± 45.02	110.41 ± 44.82	0.0053
Total cholesterol (mg/dL)	195.62 ± 40.37	195.96 ± 40.27	193.98 ± 40.82	<0.0001
Triglycerides (mg/dL)	174.79 ± 128.3	176.58 ± 129.36	166.19 ± 122.71	<0.0001
Fasting glucose (mg/dL)	143.72 ± 42.77	144.53 ± 42.62	139.83 ± 43.3	<0.0001
**Antidiabetic medication**				
Insulin only	43,166 (7.98)	31,478 (7.04)	11,688 (12.55)	<0.0001
Sulfonylurea	268,152 (49.6)	213,801 (47.78)	54,351 (58.35)	<0.0001
Metformin	273,496 (50.59)	219,130 (48.97)	54,366 (58.36)	<0.0001
Meglitinide	14,412 (2.67)	11,117 (2.48)	3,295 (3.54)	<0.0001
Thiazolidinedione	44,297 (8.19)	35,547 (7.94)	8,750 (9.39)	<0.0001
DPP-4 inhibitor	36,267 (6.71)	29,291 (6.55)	6,976 (7.49)	<0.0001
α-glucosidase inhibitor	73,325 (13.56)	56,519 (12.63)	16,806 (18.04)	<0.0001
Glucagon-like peptide-1 (GLP-1) Receptor agonists	13 (0)	11 (0)	2 (0)	0.8601

*VIM, variability independent of the mean; ASV, average successive variability; SD, standard deviation; CV, coefficient of variation; BMI: body mass index; HDL, high-density lipoprotein; LDL, low-density lipoprotein*.

### Body Weight Variability and Risk of Depression

Higher BWV was associated with a significantly higher rate of new-onset depression after multivariate adjustments (Table 2). Compared to the lowest BWV quartile group calculated by VIM, HR of depression for the highest quartile group was 1.17 (95% CI: 1.15–1.19). When analyzed by sex, males in the highest BWV quartile group showed a 22% greater risk of depression (HR: 1.22, 95% CI: 1.19–1.25) whereas females in the same quartile group showed a 12% greater risk of depression (HR: 1.12, 95% CI: 1.09–1.55) compared to the lowest quartile group.

**Table 2:** Risk of incident depression by quartiles of VIM for body weight among patients with type 2 DM^*^.

	**Total (*N*)**	**Newly onset depression (*N*)**	**Incidence rate**	**Person-years**	**HR (95% CI)**	
					**unadjusted**	**adjusted**
**Total**						
Q1	135,179	21,854	29.54	739693.03	1 (Ref.)	1 (Ref.)
Q2	135,198	21,841	29.40	742919.97	0.99 (0.98,1.01)	1.01 (0.99,1.03)
Q3	135,146	23,155	31.53	734358.86	1.07 (1.05,1.09)	1.07 (1.05,1.09)
Q4	135,070	26,299	37.00	710803.77	1.25 (1.23,1.28)	1.17 (1.15,1.19)
**Male**						
Q1	97,503	12,869	23.72	542525.54	1 (Ref.)	1 (Ref.)
Q2	95,400	12,321	23.03	534986.1	0.97 (0.95,1.00)	1.01 (0.99,1.04)
Q3	93,059	12,679	24.54	516654.31	1.04 (1.01,1.06)	1.08 (1.06,1.11)
Q4	85,817	13,168	28.49	462268.76	1.20 (1.17,1.23)	1.22 (1.19,1.25)
**Female**						
Q1	37,676	8,985	45.57	197167.49	1 (Ref.)	1 (Ref.)
Q2	39,798	9,520	45.78	207933.87	1.01 (0.98,1.03)	1.01 (0.98,1.04)
Q3	42,087	10,476	48.12	217704.55	1.06 (1.03,1.09)	1.05 (1.02,1.08)
Q4	49,253	13,131	52.83	248535.01	1.16 (1.13,1.19)	1.12 (1.09,1.15)
***p*** **for interaction**					0.0008	<0.0001

* *adjusted for age, sex, smoking status, drinking status, level of physical activity, low income, presence of medical comorbidities including hypertension, dyslipidemia, and variables related to the severity of diabetes, including insulin use, the number of oral anti-diabetic medications, and duration of diabetes. VIM, variability independent of the mean*.

### Body Weight Variability and Risk of Depression by Baseline Obesity

**Figure 2:**
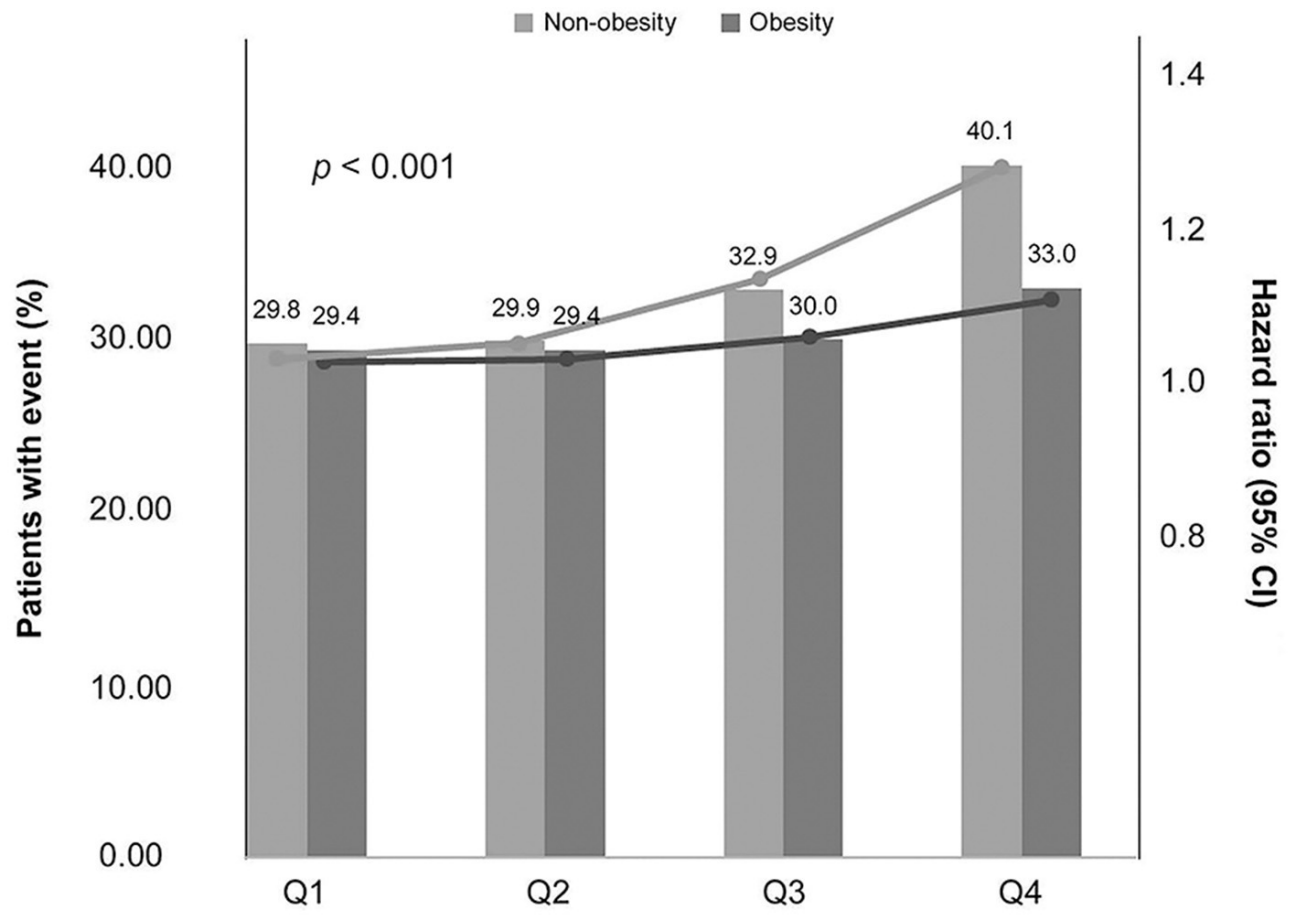
Association between the risk of depression and quartiles of BWV as a function of baseline obesity. The line represents depression incidence, and the bars represent HR. The *P* values indicate a significant difference between non-obesity and obesity in the respective BWV quartiles.

In patients with baseline obesity, higher BWV numerically increased the risk of depression. Compared to the lowest quartile, obese patients in the highest BWV quartile group had a 12% greater risk of depression per the adjusted model (HR: 1.12, 95% CI: 1.09–1.15). The association was even stronger in non-obese patients, where the risk of depression increased by 20% in the highest BWV quartile group (HR: 1.20, 95% CI: 1.17–1.23) compared to the lowest quartile group (*p* < 0.001). A steep increase of depression risk was observed in the highest BWV quartile group compared to the lower three quartiles of non-obese type 2 DM patients (Figure 2).

The probability of depression estimated by Kaplan-Meier curves according to each quartile of BWV was significantly increased in the highest quartile of both non-obese and obese patients with Type 2 DM, with the degree of increase being greater in the non-obese group (Supplementary Figure 1).

### Stratified Analysis

**Figure 3:**
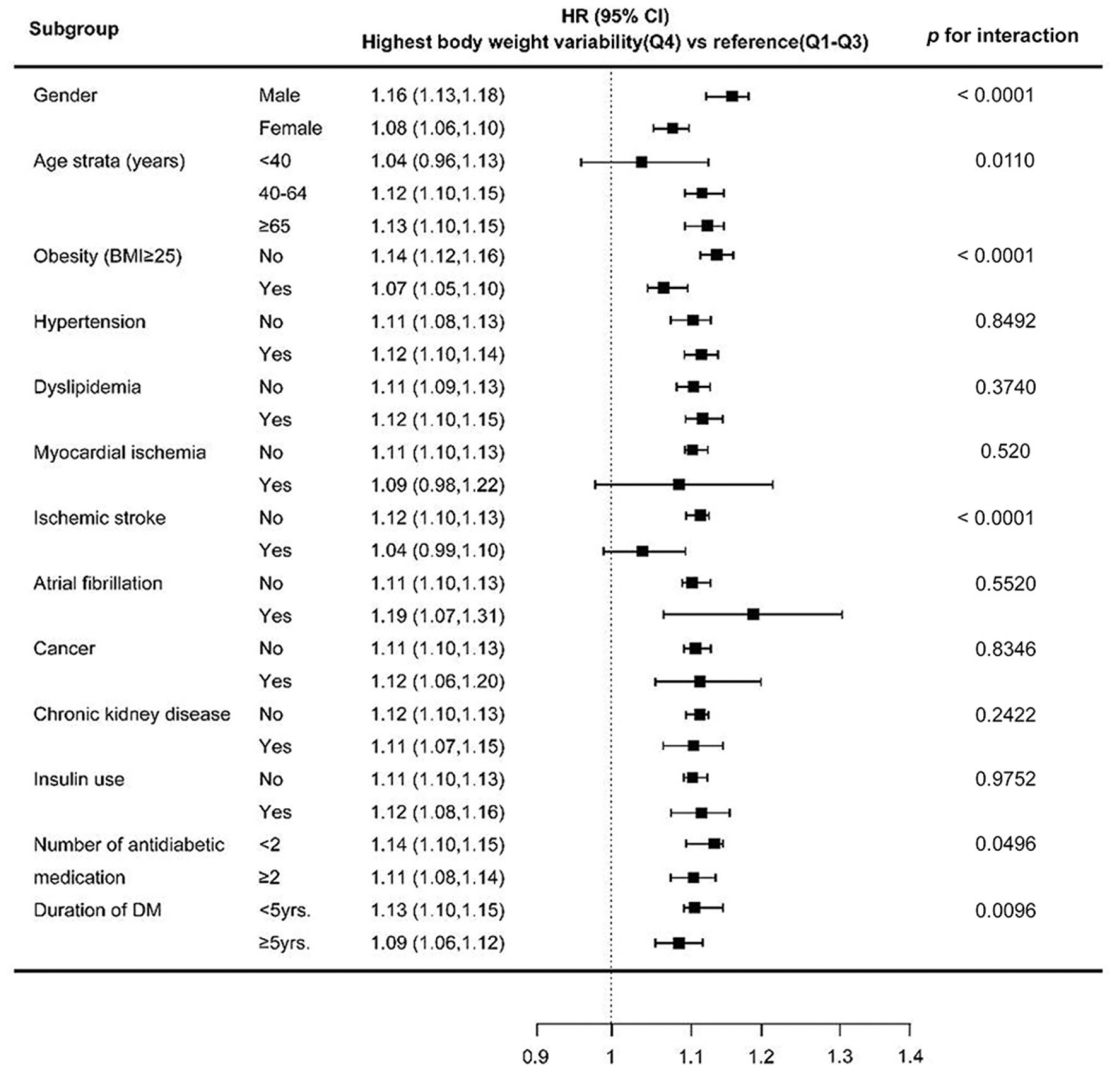
HR (95% CI) of depression in the highest quartile vs. lower three quartiles of BWV. Estimates were adjusted for age strata, gender, presence of obesity, medical comorbidities and severity of diabetes, except for the one stratified on. The horizontal lines represent 95 % CI. HR, hazard ratio; CI, confidence interval.

Figure 3 shows results of stratified analyses comparing the highest BWV quartile group to the lower three quartile groups. Higher BWV predicted increased risk of new-onset depression overall. The association was significantly stronger in males of older age, or with less severe diabetic status (i.e., use of less than two types of antidiabetic medications or shorter duration of DM) (all *p* < 0.05).

## Discussion

To the best of our knowledge, this is the first cohort study on the incidence of depression in type 2 DM patients with BWV. This study covered a large sample of a nationwide population (> 540,000 individuals) with a long follow up period of more than 7 years. We found that higher BWV was significantly associated with an increased risk of depression. This association was greater in non-obese patients than in obese patients independent of medical comorbidities. Stratified analysis also showed a consistent association between higher BWV and increased risk of depression. These findings may be considered to be less representative since the HR of depression in the highest BWV group are numerically small [i.e., 1.17 (95% CI 1.15–1.19) compared to the lowest BWV quartile group], but depression in diabetic patients is associated with increased mortality and worse prognosis so it should be interpreted in clinical significance.

The pathophysiological pathway underlying BWV and the development of depression has been poorly understood. Excessive weight fluctuation might cause alternation between a rapid shrinkage and a rapid expansion of adipose tissues. This mechanical cell stress to adipose tissues during weight fluctuation can result in accumulation of visceral fat mass and increased inflammatory responses of adipose cells, which could worsen vascular pathology and subsequently increase the risk of cardiovascular outcomes of patients with diabetes (15, 29). A recent study has demonstrated the association between body fat mass and depression (30), suggesting that inflammatory response during fat accumulation might also play an important role in the development of depression. Inflammatory cytokines such as tumor necrosis factor, C-reactive protein, interleukin (IL)-1b, and IL-6 are increased in depressed individuals (31). Several studies have also found that increased inflammation is associated with decreased connectivity of the dorsal/ventral striatum and ventromedial prefrontal cortex, brain regions involved in motivation and mood regulation (32, 33). Such deterioration of vascular pathology and decreased brain function caused by inflammation might have a synergistic effect on the development of depression in type 2 DM patients. Further molecular level research is needed to demonstrate the mechanisms involved in BWV-induced inflammation in depressed diabetic patients.

Another possible explanation is that endocrine factors (such as adipokines including leptin, adiponectin, and resistin, secreted by adipose tissues) might be involved in the effect of BWV on depression. Endocrine molecules, especially leptin and adiponectin, are known to be associated with depression by regulating the hypothalamic-pituitary-adrenal (HPA) axis and neuronal plasticity in the brain (34, 35). Adipose cell stress resulting from BWV could cause changes in blood levels of such adipokines, thus affecting mood regulation. Further research on such biological mechanisms is necessary.

The effect of high BWV on the development of depression in type 2 DM was greater in non-obese patients than in obese patients. Most studies examining BWV of DM patients showed a higher risk of medical complications including cardiovascular events and mortality in obese patients than in non-obese patients (3, 5, 36). Although comparative studies are lacking, psychosocial components could have more effects on the development of depression than other medical complications in type 2 DM patients. One explanation is that non-obese patients might react more sensitively to BWV than obese patients who frequently experience it because BWV is usually not a major problem for non-obese patients (whether the change is intentional or not) (37). It has been shown that body weight perception, not just actual weight itself, is associated with unhealthy weight control behaviors and depression (38). In addition, it has been increasingly reported that, besides obesity, underweight status is also associated with neuroticism, low life satisfaction, and depressed mood (39, 40). As such, BWV is not simply a metabolic consequence. Various psychological and behavioral factors might be involved in BWV. This supports our finding that the effect of BWV on depression is not limited to obese patients. Further study is needed the phenomenon of “obesity paradox” (41) is occurring psychologically as a flexible acceptance of body changes. Additionally, although the prescription rate was not high in our study cohort, glucagon-like peptide 1-receptor agonist or sodium glucose cotransporter 2-inhibitor can reduce weight during long-term use among diabetic agents, so it could be considered when interpretating higher depression risk in low BMI patients at baseline.

Also, BWV could be a consequence but not a cause of subclinical depression or other physical illness. That said, we performed several stratified analyses to minimize the effect of other causes of illness as much as possible.

Female gender is a demonstrated risk factor for developing depression. Several cross sectional studies have suggested an increased depression-obesity association in females (42). In our study as well, the prevalence of depression was higher in females. However, BWV–associated risk of depression was stronger in males.

Previous studies have mostly focused on the association between advanced stage of diabetes (more oral antidiabetic medication or longer duration of DM) and increased risk of depression in type 2 DM patients with obesity (43, 44). However, we found a reverse association between the severity of diabetes and depression in type 2 patients with BWV (i.e., the risk of depression was higher in less severe diabetic status). Although the mechanism is currently unclear, depression caused by BWV seems to have a different mechanism from depression caused by obesity in patients with type 2 DM. In addition, since men with less severe diabetes have a lower risk of depression compared to women with advanced diabetes, the relative risk of depression caused by BWV might be greater in these groups. Further research is warranted to confirm the background mechanisms and should more focus on several antidiabetic medications on the risk of depression [such as metformin, which is known to be associated with decreased rate of incident depression in recent study (45)] in time and dose dependent manner.

Several limitations should be considered when interpreting our results. First, the diagnosis of depression was based on claim data, and therefore some degree of misclassification is possible. Second, whether weight change was intended or naturally occurring was unknown. Third, restriction of the analysis to Koreans reduces generalizability. Fourth, as in any retrospective study, reverse causality might be overlooked. Therefore, we set a 2 year lag period and excluded previous depression diagnosis to avoid reverse causation as much as possible. Further study is needed to consider causality in prospective design. Fifth, variability independent of the mean (VIM) was used as the primary measure of BWV and as this is a mathematical estimation clinical relevance should be considered in interpretation.

## Conclusions

This large cohort study confirmed that BWV was significantly associated with an increased risk of depression in patients with type 2 DM. The magnitude of depression risk was greater in those who were not obese at baseline. Thus, BWV may serve as an indicator for early detection of depression in type 2 DM patients.

## Data Availability Statement

The raw data supporting the conclusions of this article will be made available by the authors, without undue reservation.

## Ethics Statement

The studies involving human participants were reviewed and approved by the Institutional Review Board (IRB) of Samsung Medical Center in Seoul, Korea (IRB No: 2020-10-034). Written informed consent for participation was not required for this study in accordance with the national legislation and the institutional requirements.

## Author Contributions

JHA participated in the study design, conception, data analysis, wrote the first manuscript drafting, and revised new drafts from co-authors. K-dH, J-HJ and JY participated in directed acquisition of the data and data analysis. MF, DM, S-MJ, DWS and KYH conceptualized the study and revised the manuscript. HJJ participated in whole study design and conception, and manuscript drafting. All authors read and approved the final manuscript.

## Funding

This research was supported by a grant of the Korea Health Technology R&D Project through the Korea Health Industry Development Institute (KHIDI), funded by the Ministry of Health & Welfare, Republic of Korea (Grant Number: HI21C0885), and by the Healthcare AI Convergence Research & Development Program (No. S1601-20-1041) through the National IT Industry Promotion Agency of Korea (NIPA) funded by the Ministry of Science and ICT.

## Conflict of Interest

DM has received research support from Nordic Naturals and Heckel Medizintechnik GmbH. He also works with the MGH Clinical Trials Network and Institute (CTNI), which has received research funding from multiple pharmaceutical companies and NIMH. The remaining authors declare that the research was conducted in the absence of any commercial or financial relationships that could be construed as a potential conflict of interest.

## Publisher's Note

All claims expressed in this article are solely those of the authors and do not necessarily represent those of their affiliated organizations, or those of the publisher, the editors and the reviewers. Any product that may be evaluated in this article, or claim that may be made by its manufacturer, is not guaranteed or endorsed by the publisher.
