# Supplementary material for: High Bodyweight Variability Increases Depression Risk in Patients With Type 2 Diabetes Mellitus: A Nationwide Cohort Study in Korea
Source: Front Psychiatry. 2021 Dec 10;12:765129. doi: 10.3389/fpsyt.2021.765129 (PMC8702997; doi:10.3389/fpsyt.2021.765129)
Supplement: Supplementary file 1 [file Data_Sheet_1.docx]

**
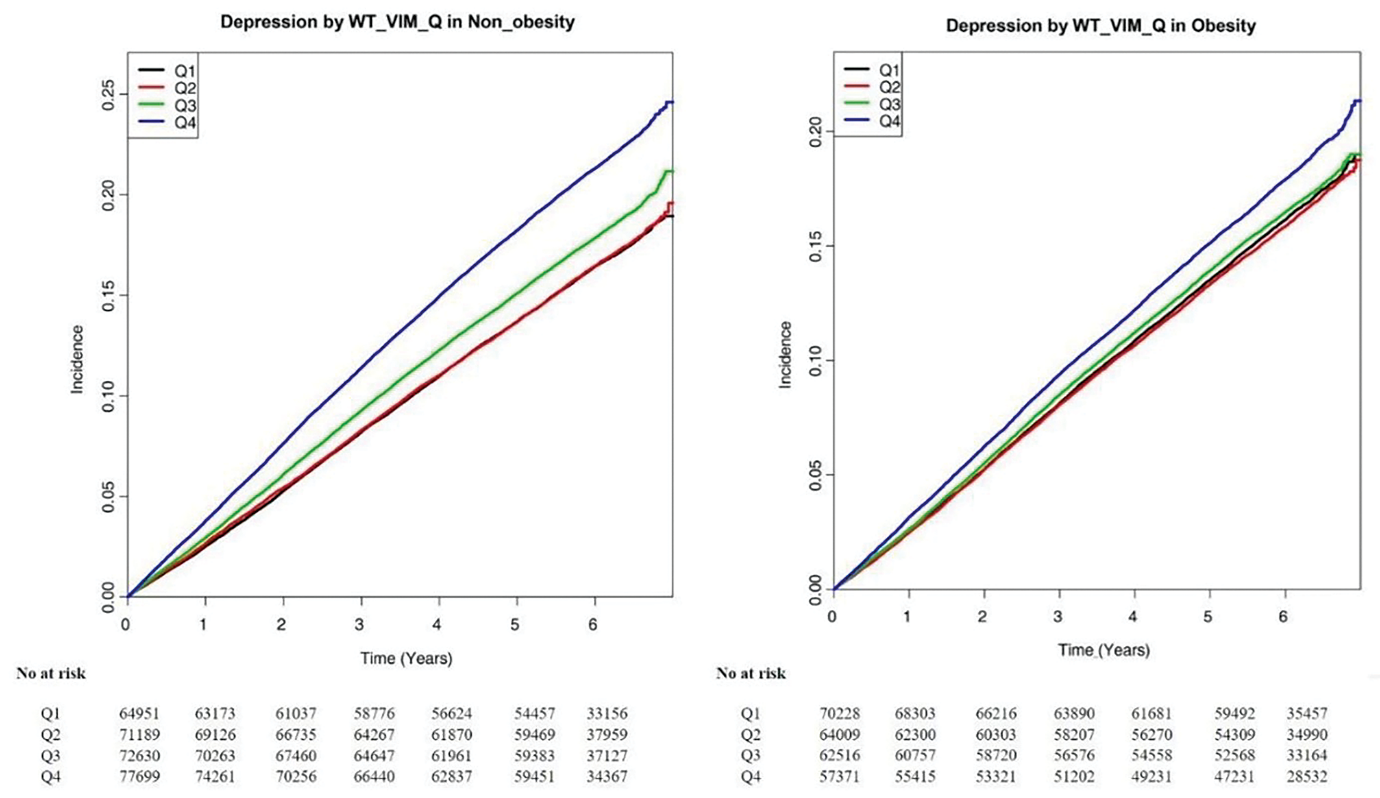
**

**Supplementary Figure S1 |** Time-to-event curve for depression according to BWV quartile in non-obese and obese groups of patients with type 2 DM.
